# Sibling risks in cancer: clues to recessive or X-linked genes?

**DOI:** 10.1054/bjoc.2000.1585

**Published:** 2001-02

**Authors:** K Hemminki, P Vaittinen, C Dong, D Easton

**Affiliations:** Department of Biosciences at Novum, Karolinska Institute, 141 57 Huddinge, Sweden; CRC Genetic Epidemiology Unit, Strangeways Research Laboratories, Worts Causeway, Cambridge, CB1 8RN, UK

**Keywords:** familial cancer, liability, heredity, cancer modelling, genetic epidemiology

## Abstract

A systematic analysis of cancer risks to offspring and to siblings of cancer cases was carried out based on the nation-wide Swedish Family-Cancer Database. For all 13 cancer sites examined, risks to both offspring and siblings of cases of cancer at the same site were significantly elevated. The relative risk to siblings was approximately 2 fold more than the offspring risk for cancers of the prostate, testis, kidney and bladder, suggesting that recessive or X-linked susceptibility genes may be important for these cancers. Risks to siblings of cases where a parent was also affected were increased >20 fold over population rates for colorectal, ovarian, prostate and renal cancer, and for leukaemia, consistent with the effects of rare high-risk susceptibility alleles. © 2001 Cancer Research Campaign http://www.bjcancer.com


*British Journal of Cancer* (2001) **84**(3), 388–391
© 2001 Cancer Research Campaign
doi: 10.1054/ bjoc.2000.1585, available online at http://www.idealibrary.com on IDEAL®

### Sibling risks in cancer: clues to recessive or X-linked genes?

**K Hemminki[1], P Vaittinen[1], C Dong[1] and D Easton[2]**

[1]Department of Biosciences at Novum, Karolinska Institute, 141 57 Huddinge, Sweden; [2]CRC Genetic Epidemiology Unit, Strangeways Research Laboratories, Worts Causeway, Cambridge CB1 8RN, UK

**Summary** A systematic analysis of cancer risks to offspring and to siblings of cancer cases was carried out based on the nation-wide Swedish Family-Cancer Database. For all 13 cancer sites examined, risks to both offspring and siblings of cases of cancer at the same site were significantly elevated. The relative risk to siblings was approximately 2 fold more than the offspring risk for cancers of the prostate, testis, kidney and bladder, suggesting that recessive or X-linked susceptibility genes may be important for these cancers. Risks to siblings of cases where a parent was also affected were increased >20 fold over population rates for colorectal, ovarian, prostate and renal cancer, and for leukaemia, consistent with the effects of rare high-risk susceptibility alleles. © 2001 Cancer Research Campaign http://www.bjcancer.com

**Keywords**: familial cancer; liability; heredity; cancer modelling; genetic epidemiology

Genetic epidemiological studies have been instrumental in guiding efforts to map disease susceptibility genes, firstly, by identifying diseases with marked familial aggregation and, secondly, by guiding the selection of appropriate families for linkage or mutation analysis. This approach has been successful in, for example, breast cancer where segregation analysis suggested the presence of a high-risk, low-frequency allele that explained a part of the familial aggregation of the disease, eventually confirmed by the identification of *BRCA1* and *BRCA2* (Szabo and King, 1997). For prostate cancer, segregation analysis suggested dominant pattern of inheritance but the higher prostate cancer risks between affected brothers than father–son pairs suggested the presence of a recessive or X-linked component (Monroe et al, 1995). Both autosomal and X-linked prostate cancer susceptibility loci have been mapped by linkage (Narod, 1999). A putative X-linked testis cancer susceptibility locus has also recently been mapped (Rapley et al, 2000). Even though many systematic studies have been carried out on familial aggregation of cancer (Goldgar et al, 1994; Carstensen et al, 1996; Easton et al, 1996; Peto et al, 1996; Hemminki et al, 1998), specific analysis of risks to siblings has been limited to studies on particular cancer sites and only some of the commonest cancers have been examined (Carstensen et al, 1996; Easton et al, 1996; Peto et al, 1996). Risks to siblings are of interest since high risks, in comparison with risks to parents or offspring, may indicate recessive or X-linked components to the disease.

Here we use the nation-wide Swedish Family-Cancer Database on 9.6 million individuals and more than 700 000 primary cancers to assess systematically the offspring and sibling risks for common cancers. The family dataset is unique in size, coverage and longitudinal time span.

*Received 7 June 2000*
*Revised 16 October 2000*
*Accepted 16 October 2000*

*Correspondence to:* K Hemminki

### SUBJECTS AND METHODS

The Swedish Family-Cancer Database includes persons born after 1934 with their biological parents (Hemminki and Vaittinen, 1998; Hemminki et al, 1998). Cancers were retrieved from the nation-wide Swedish Cancer Registry covering the period 1958 to 1996; thus, the maximum age at last follow-up for offspring was 61. A 4-digit diagnostic code according to the 7th revision of the International Classification of Diseases (ICD-7) was used. In the tables, the category ‘colorectal’ is defined by ICD-7 codes 153 and 154.0 (i.e. excluding anus) and ‘lung’ by codes 162 and 163.

Age-specific incidence rates to offspring of affected parents (‘offspring risks’), were calculated in 5-year diagnosis age bands from 10 to 61 years. Standardized incidence ratios (SIRs) were calculated as the ratio of observed (O) to expected (E) number of cases. The expected numbers were calculated from age-, sex- and tumour type-standardized rates (Esteve et al, 1994). Confidence intervals (95% CI) were calculated assuming a Poisson distribution (Esteve et al, 1994).

Risks to full siblings of affected individuals were calculated in a similar manner; however, because individuals may have more than one affected sibling, risks to siblings may be defined in several ways. We considered two definitions, referred to as the ‘cohort method’ and the ‘multiple-counting method’. In the cohort method one simply defines a cohort of individuals with at least one affected sibling, and computes the incidence rates in this cohort over the period 1958 to 1996 as before. Note that in a family with two or more affected siblings, each affected individual is included in the cohort (as the sibling of an affected individual). In the multiple-counting method by contrast, one considers all possible pairs of siblings, and computes the incidence in a ‘cohort’ of individuals whose co-sibling is affected. The methods are formally identical for families with at most two siblings, and for families with only one affected individual. However, under the multiple counting method, an individual with $n$ affected siblings will be included $n$ times in the cohort, rather than once in the cohort method. Thus the incidence rates in the cohort method are given by the formula:

$$\frac{\sum_{k=2}^{N} n_k}{\left[\sum_{k=1}^{N} p_k + \sum_{k=2}^{N} y_k\right]}$$

And in the multiple counting method by the formula:

$$\frac{\sum_{k=2}^{N} (k\text{-}1) n_k}{\left[\sum_{k=1}^{N} k p_k + \sum_{k=2}^{N} (k-1) y_k\right]}$$

Where $n_k$ is the number of affected individuals with $k$ affected siblings, $p_k$ the number of person-years contributed by unaffected individuals in families with $k$ affected siblings, and $y_k$ the number of person-years contributed by affected siblings in families with $k$ affected siblings, in the relevant age/sex/period category. The corresponding reference rates (for both methods) are given by:

$$\frac{\sum_{k=1}^{N} n_k}{\left[\sum_{k=0}^{N} p_k + \sum_{k=1}^{N} y_k\right]}$$

Although more complex, the multiple counting method has the theoretical advantage that it provides an unbiassed estimate of the risk to the siblings of a specified affected individual, regardless of the family size distribution (provided that risks are not related to family size), and that this parameter will be equal to the offspring risk under any additive genetic models (and, to good approximation, under dominant models). Under the cohort method, the relative risks would be expected to decrease with family size, and can be slightly lower than the offspring risks under additive models. In practice, however, we found that the relative risk estimates given by the two methods were almost identical, and only those given by the cohort method are shown. An additional complication in the computation of risks to siblings is that, since it is based on complete ascertainment of sibships with affected individuals, families with multiple affected individuals are ascertained multiple times, so that the observed cancers in the cohort are not independent. Thus a family with 2 affected siblings will contribute 2 observed cancers, whereas it is in fact only one independent event, leading to an inflated variance of the relative risk. To correct for this, we approximated the variance of the log (relative risk) by 1/(N-M), rather than by 1/N, where N is the total number of cancers and M is the number of ascertained families (Goldgar et al, 1994).

### RESULTS

Table 1 shows offspring and sibling risks for 13 common cancer sites. The SIRs for both offspring and sibling risks were significantly greater than 1 for all cancer sites. The offspring risks range from 4.09 for testis to 1.55 for urinary bladder. The sibling risks were greater than the offspring risks for 12 of the 13 sites, the sole exception being ovarian cancer. The difference in risk reached statistical significance for cancers of the colorectum ($P < 0.001$), breast ($P = 0.024$), prostate ($P = 0.003$), testis ($P = 0.04$) and kidney ($P = 0.012$), and for melanoma ($P = 0.019$) and leukaemia ($P = 0.042$). After ovary, breast cancer had the smallest sibling:offspring risk ratio (1.15). The ratio was highest for kidney (2.55), testis (2.22), bladder (2.01) and prostate (1.95). There is no statistically significant sex difference (i.e., 95% CIs overlapped for SIRs of male and female offspring) for any of the comparisons shown in Table 1 but the largest difference was noted for bladder cancer. Among the 12 affected sibs, only one was female, giving a SIR of 4.06 (95% CI 2.02–7.30) for male offspring and 1.07 (0–6.17) for female offspring.

For sibling risks we separated those who had an affected parent in order to identify possible rare dominant gene effects. Higher SIRs were observed in siblings with an affected parent, compared with those with unaffected parents, for all sites except cervix, testis and bladder where no affected sibling pairs with an affected parent were present. SIRs were particularly high for colorectal cancer (20.46), ovary (31.10), prostate (31.35), kidney (28.90) and leukaemia (32.08).

### DISCUSSION

The observed excess risks of cancer in siblings could be due to either genetic or environmental causes, or both. We have assessed the effect of shared environment in adulthood from this Database by comparing cancer risks between spouses (Hemminki and Dong, 2000; Hemminki et al, 2000). Among the cancers discussed here, significant spouse concordance was only found for stomach and lung cancers, and melanomas if diagnosed at a young age. Siblings, however, share lifestyle risk factors in childhood and this may be a cause of some of the increased sibling risks, as compared with offspring risk. In a recent twin analysis by Lichtenstein et al (2000), based on a combined cohort of twins from Sweden, Denmark and Finland, risks to monozygotic (MZ) twins of cancer cases were more than twice the risk to dizygotic (DZ) twins for prostate, ovarian and bladder cancer, although the numbers (except for prostate cancer) were small. If one assumes that the distribution of shared environmental factors will be similar in MZ and DZ twins, this suggests that environmental factors are unlikely to be the main determinants of familial risk, at least for these cancer sites.

Another possible factor leading to higher sibling relative risks is that some familial relative risks are age-dependent. In this study, all affected siblings must be diagnosed under age 61, whereas the affected parents used to determine offspring risks may be older. If familial relative risks decline with age, the sibling relative risks will be higher than offspring risks even if the age-specific risks are equal. This may be part of the explanation for the effects of colorectal and prostate cancer. At present, there are too few data to allow for the age-specific effects adequately in this cohort.

If the higher sibling risks are genetic, they could be due either to autosomal recessive or X-linked recessive genes. An X-linked susceptibility allele would be predicted to cause an increased risk only in male siblings of male cases, whereas autosomal recessive alleles affect both sexes. In none of the previous analysis of offspring–parents risks from this Database nor in the present analysis have we observed significant sex differences in familial relative risks in the cancers that affect both genders, with the possible exception of bladder cancer noted above, suggesting that X-linked recessive effects are likely to be weak. In male-specific cancers of testis and prostate, it would not be possible to distinguish autosomal and X-linked recessive patterns in 2-generation families.

© 2001 Cancer Research Campaign 

**Table 1** Offspring and sibling risks for concordant cancers in families

| Cancer site | Offspring risk | | | | | Parent with same cancer | Sibling risk | | | | | Sibling: Offspring |
|---|---|---|---|---|---|---|---|---|---|---|---|---|
| | O | E | SIR | 95% CI | | | O | E | SIR | 95% CI | | |
| | | | | | | No | 38 | 14.0 | 2.71 | 1.73 | 4.25 | 1.47 |
| Colorectum | | | | | | Yes | 30 | 1.5 | 20.46 | 12.33 | 33.94 | |
| | 429 | 232 | 1.85 | 1.67 | 2.03 | Total | 68 | 15.5 | 4.39 | 3.14 | 6.14 | |
| | | | | | | No | 11 | 4.5 | 2.46 | 1.02 | 5.91 | 1.47 |
| Lung | | | | | | Yes | 3 | 0.3 | 11.07 | 1.56 | 78.59 | |
| | 125 | 74.4 | 1.68 | 1.40 | 2.00 | Total | 14 | 4.7 | 2.96 | 1.41 | 6.21 | |
| | | | | | | No | 419 | 199.9 | 2.10 | 1.83 | 2.40 | 1.15 |
| Breast | | | | | | Yes | 42 | 14.6 | 2.88 | 1.88 | 4.41 | |
| | 1512 | 828 | 1.83 | 1.73 | 1.92 | Total | 461 | 214.5 | 2.15 | 1.89 | 2.45 | |
| | | | | | | No | 28 | 12.4 | 2.27 | 1.34 | 3.83 | 1.23 |
| Cervix | | | | | | Yes | 0 | 0.3 | | | | |
| | 86 | 46.5 | 1.85 | 1.48 | 2.28 | Total | 28 | 12.6 | 2.22 | 1.31 | 3.75 | |
| | | | | | | No | 12 | 3.0 | 3.94 | 1.77 | 8.77 | 1.65 |
| Endometrium | | | | | | Yes | 0 | 0.1 | | | | |
| | 69 | 28.9 | 2.39 | 1.86 | 3.03 | Total | 12 | 3.1 | 3.82 | 1.72 | 8.50 | |
| | | | | | | No | 12 | 6.6 | 1.83 | 0.82 | 4.07 | 0.66 |
| Ovary | | | | | | Yes | 4 | 0.1 | 31.10 | 7.78 | 124.4 | |
| | 95 | 34.3 | 2.77 | 2.24 | 3.38 | Total | 16 | 6.7 | 2.39 | 1.20 | 4.78 | |
| | | | | | | No | 4 | 0.8 | 5.00 | 1.25 | 19.99 | 1.95 |
| Prostate | | | | | | Yes | 6 | 0.2 | 31.35 | 10.11 | 97.20 | |
| | 167 | 65.2 | 2.56 | 2.18 | 2.98 | Total | 10 | 1.0 | 10.09 | 4.20 | 24.24 | |
| | | | | | | No | 38 | 4.2 | 9.07 | 5.79 | 14.22 | 2.22 |
| Testis | | | | | | Yes | 0 | 0.0 | | | | |
| | 12 | 2.9 | 4.09 | 2.13 | 7.20 | Total | 38 | 4.2 | 9.05 | 5.77 | 14.19 | |
| | | | | | | No | 10 | 2.3 | 4.38 | 1.82 | 10.52 | 2.54 |
| Kidney | | | | | | Yes | 2 | 0.1 | 28.90 | 4.07 | 205.2 | |
| | 45 | 26.2 | 1.72 | 1.25 | 2.30 | Total | 12 | 2.4 | 5.10 | 2.29 | 11.35 | |
| | | | | | | No | 12 | 3.9 | 3.11 | 1.40 | 6.92 | 2.01 |
| Bladder | | | | | | Yes | 0 | 0.1 | | | | |
| | 69 | 44.6 | 1.55 | 1.20 | 1.96 | Total | 12 | 4.0 | 3.02 | 1.36 | 6.72 | |
| | | | | | | No | 107 | 30.0 | 3.56 | 2.72 | 4.66 | 1.37 |
| Melanoma | | | | | | Yes | 7 | 0.6 | 11.12 | 3.59 | 34.48 | |
| | 189 | 72.6 | 2.60 | 2.24 | 3.00 | Total | 114 | 30.7 | 3.72 | 2.86 | 4.83 | |
| | | | | | | No | 34 | 14.4 | 2.36 | 1.47 | 3.80 | 1.47 |
| Lymphoma | | | | | | Yes | 2 | 0.3 | 6.93 | 0.98 | 49.20 | |
| | 106 | 66.2 | 1.60 | 1.31 | 1.94 | Total | 36 | 14.7 | 2.45 | 1.54 | 3.89 | |
| | | | | | | No | 24 | 8.2 | 2.93 | 1.66 | 5.16 | 1.59 |
| Leukaemia | 61 | 33.1 | 1.84 | 1.4 | 2.37 | Yes | 4 | 0.1 | 32.08 | 8.02 | 28.3 | |
| | | | | | | Total | 28 | 8.3 | 3.37 | 2.00 | 5.69 | |

For most of the cancer sites, the sibling risks were substantially higher when a parent was also affected. This effect was particularly marked for colorectal, ovarian, prostate and kidney cancer, and for melanoma and leukaemia, although all these observations, except for colorectal cancer, are based on small numbers. These high risks are consistent with the effects of a highly penetrant dominant susceptibility gene responsible for a proportion of cases. The observed familial risks for ovarian cancer are mostly due to mutations in BRCA1 and BRCA2 (Antoniou et al, 2000). Interestingly, the high relative risk in siblings with an affected mother is not observed for breast cancer, consistent with previous observations that BRCA1/2 make only a minor contribution to familial breast cancer (Peto et al, 2000).

The sibling:offspring risk ratio was close to 1 for both ovarian and breast cancer, suggesting that the main genetic effects are dominant or additive. For these cancers our sibling and offspring risks are in line with comprehensive meta-analyses of the published literature (Pharoah et al, 1997; Stratton et al, 1998). For colorectal, lung and endometrial cancer, and for lymphoma and leukaemia, the ratio was around 1.5, suggesting some recessive or X-linked component. At the other extreme kidney, bladder, testis and prostate cancer show a high sibling:offspring risk ratio. The high sibling risk for prostate cancer is consistent with the observation that some multiple case prostate cancer families show linkage to a region on chromosome Xq, while other families appear to be linked to autosomal loci. (Xu et al, 1998; Grönberg et al, 1999; Narod, 1999). A particularly high risk to MZ twins of prostate cancer cases was observed by Lichtenstein et al (2000) (relative risk 12.3 vs 3.1 for DZ twins), which might suggest a recessive component. An X-linked locus has also recently been mapped for testicular cancer (Rapley et al, 2000). The most important susceptibility gene for kidney cancer is the VHL gene, responsible for the von Hippel Lindau syndrome (Fearon, 1997); this, however, is unlikely to be a major determinant of familial kidney cancer. No major susceptibility genes for bladder cancer have yet been identified, although mutations in the mismatch repair genes, Rb and p16 are associated with some increased risk.

 *© 2001 Cancer Research Campaign*

In conclusion, these results raise the possibility that recessive or X-linked susceptibility genes, as yet unidentified, may be important for some common cancers. Such genes will not be mappable by linkage studies in large families, but may be identifiable through large series of affected sibling pairs or association studies.

### ACKNOWLEDGEMENTS

The work was supported by The Cancer Fund.

### REFERENCES

Ahlbom A, Lichtenstein P, Malmström H, Feychting M, Hemminki K and Pedersen NL (1997) Cancer in twins: genetic and nongenetic familial risk factors. *J Natl Cancer Inst* **89**: 287–293

Antoniou AC, Gayther SA, Stratton JF, Ponder BAJ and Easton DF (2000) Risk models for familial ovarian and breast cancer. *Genet Epidemiol* **18**: 173–190

Cairns P and Sidransky D (1998) Bladder cancer. In: Vogelstein B and Kinzler K (eds) *The Genetic Basis of Human Cancer,* pp. 639–645. McGraw-Hill: New York

Carstensen B, Soll-Johanning H, Villadsen E, Söndergaard J and Lynge E (1996) Familial aggregation of colorectal cancer in the general population. *Int J Cancer* **68**: 428–435

Claus E, Schildkraut J, Iversen E, Berry D and Parmigiani G (1998) Effect of BRCA1 and BRCA2 on the association between breast cancer risk and family history. *J Natl Cancer Inst* **90**: 1824–1829

Easton D, Matthews F, Ford D, Swerdlow A and Peto J (1996) Cancer mortality in relatives of women with ovarian cancer: the OPCS study. *Int J Cancer* **65**: 284–294

Esteve J, Benhamou E and Raymond L (1994) *Statistical Methods in Cancer Research*, Vol. 128. IARC Scientific Publication. IARC: Lyon

Fearon ER (1997) Human cancer syndromes: clues to the origin and nature of cancer. *Science* **278**: 1043–1050

Goldgar DE, Easton DF, Cannon-Albright LA and Skolnick MH (1994) Systematic population-based assessment of cancer risk in first-degree relatives of cancer probands. *J Natl Cancer Inst* **86**: 1600–1607

Grönberg H, Smith J, Emanuelsson M, Jonsson B-A, Bergh A, Carpten J, Isaacs W, Xu J, Meyers D, Trent J and Dambert JE (1999) In Swedish families with hereditary prostate cancer, linkage to the HPC1 locus on chromosome 1q24–25 is restricted to families with early-onset prostate cancer. *Am J Hum Genet* **65**: 134–140

Hemminki K and Vaittinen P (1998) National database of familial cancer in Sweden. *Genet Epidemiol* **15**: 225–236

Hemminki K, Vaittinen P and Kyyrönen P (1998) Age-specific familial risks in common cancers of the offspring. *Int J Cancer* **78**: 172–175

Hemminki K and Dong C (2000) Life style and cancer: protection form a cancer-free spouse. *Int J Cancer* **87**: 308–309

Hemminki K, Storwall I, Vaittinen P and Lichtenstein P (2000) Estimation of genetic and environmental components in colorectal and lung cancer and melanoma. *Genet Epidemiol* (in press)

IARC (1997) *Cancer Incidence in Five Continents*, Parkin D, Whelan S, Ferlay J, Raymond L and Young S (eds), Vol. VII. IARC: Lyon.

Kumar V, Cotran R and Robbins S (1997) *Basic Pathology* WB Saunders: Philadelphia

Lichtenstein P, Holm N, Verkasalo P, Illiado A, Kaprio J, Koskenvuo M, Pukkala E, Skytthe A and Hemminki K (2000) Environmental and heritable components of cancer: joint analyses of three Nordic twin cohorts. *N Engl J Med* **343**: 78–85

Monroe K, Yu M, Kolonel L, Coetzee G, Wilkens L, Ross R and Henderson B (1995) Evidence of an X-linked or recessive genetic component to prostate cancer risk. *Nature Med* **1**: 827–829

Narod S (1999) Genetic epidemiology of prostate cancer. *Biochim Biophys Acta* **1423**: F1–13

Peto J, Easton D, Matthews F, Ford D and Swerdlow A (1996) Cancer mortality in relatives of women with breast cancer: the OPCS study. *Int J Cancer* **65**: 275–283

Peto J, Collin N, Barfoot R, Seal S, Warren W, Rahman N, Easton DF, Evans C, Deacon J and Stratton MR (1999) Prevalence of BRCA1 and BRCA2 gene mutations in patients with early-onset breast cancer. *J Natl Cancer Inst* **91**: 943–949

Pharoah P, Day N, Duffy S, Easton D and Ponder B (1997) Family history and the risk of breast cancer: a systematic review and meta-analysis. *Int J Cancer* **71**: 800–809

Rapley E, Crockford G, Teare D, Biggs P, Seal S, Barfoot R, Edwards S, Hamoudi R and al e (2000) Localization to Xq27 of a susceptibility gene for testicular germ-cell tumours. *Nature Genet* **24**: 197–200

Stratton J, Pharoah P, Smith S, Easton D and Ponder B (1998) A systematic review and meta-analysis of family history and risk of ovarian cancer. *Br J Obstet Gynaecol* **105**: 493–499

Szabo CI and King MC (1997) Population genetics of BRCA1 and BRCA2 [editorial; comment]. *Am J Hum Genet* **60**: 1013–1020

Vogel F and Motulsky A (1996) *Human Genetics: problems and approaches* Springer: Heidelberg

Xu J, Meyers D, Freije D, Isaac S, Wiley K, Nusskern D, Ewing C and et al (1998) Evidence for a prostate cancer susceptibility locus on the X chromosome. *Nat Genet* **20**: 175–179

*© 2001 Cancer Research Campaign*
